# Attitudes Towards Evidence-Based Practice of Professionals Working with Children and Adolescents with Autism Spectrum Disorder in Bangladesh

**DOI:** 10.1007/s10488-022-01205-2

**Published:** 2022-06-30

**Authors:** Maleka Pervin, York Hagmayer

**Affiliations:** 1 Department of Psychology, University of Dhaka, Dhaka, Bangladesh; 2 Institute of Psychology, Georg August University of Göttingen, Göttingen, Germany

**Keywords:** Mental health professionals, Attitudes, Evidence-based practice, Autism spectrum disorder

## Abstract

Like in many lower-middle-income countries (LMIC), progress in implementing evidence-based practices (EBPs) for children with autism spectrum disorder (ASD) has been slow in Bangladesh. This cross-sectional study examined professionals’ attitudes towards evidence-based practice (EBP) for children and adolescents with ASD and explored how providers’ demographic factors are related to attitudes to and adoption of EBPs in Bangladesh. The sample consisted of 150 mental health professionals and special teachers from the urban area of Dhaka. Attitudes were assessed by the Evidence-based Practice Attitude Scale-36. Findings indicated that professionals have favorable attitudes towards EBP. Their attitudes varied depending on service settings (public clinical, private clinical, and special school) and caseload per year. Professionals who work in private and special school settings claimed to be more willing to adopt an EBP when required and perceived a higher fit of EBPs and their work than those in public clinical settings. The number of different EBPs used also differed by service setting. Every type of intervention (except medication) was used by more professionals in special schools than in private and public clinical settings. Many professionals reported few barriers to the implementation of EBPs. These findings indicate conditions that are often conducive to the implementation of EBPs. However, these results do not reflect the situation in rural areas, in which poverty is more widespread and the number of specialized professionals is low.

## Introduction

Autism Spectrum Disorder (ASD) is a neurodevelopmental disorder characterized by impairments in social communication and interaction as well as restricted and repetitive patterns of behavior (DSM-5: American Psychiatric Association, 2013; ICD-11: World Health Organization, 2019). The symptoms of ASD emerge during the first three years of life and exist throughout the life cycle (Jackson & Volkmar, 2019; Lai & Baron-Cohen, 2015; Varcin & Jeste, 2017). Individuals with autism have difficulty interacting with others: building relationships, using language, regulating their emotions, and understanding others’ points of view.

Recently, many epidemiological studies reported a prevalence of ASD that is higher than previously thought. Worldwide, there is an increasing number of children who meet the diagnostic criteria for ASD. It is estimated that approximately 1.5% of children in Europe (Germany, Poland, France, Belgium, Denmark, Iceland, Sweden, Ireland), China, and North America can be diagnosed with ASD, but the rates vary considerably between regions and populations (Bachmann et al., 2018; Boilson et al., 2016; Dereu et al., 2010; Idring et al., 2015; Lyall et al., 2017; Parner et al., 2011; Saemundsen et al., 2013; Skonieczna-Żydecka et al., 2017; Van Bakel et al., 2015). The prevalence of ASD in the U.S., for example, is 1.9%, with boys being four times more likely to be diagnosed than girls (Maenner et al., 2020; Shaw et al., 2020).

The prevalence rate in low-income countries (LIC) and lower middle-income countries (LMIC) is rather uncertain due to a lack of awareness, limited knowledge, and scarcity of well-conducted scientific studies (Samms-Vaughan, 2014). A systematic review regarding South Asia reported a prevalence ranging from 0.09% in India to 1.07% in Sri Lanka. For Bangladesh, numbers ranged from 0.15 to 0.8% (Hossain et al., 2017). According to a survey conducted by the Ministry of Health, Family, and Welfare of the Peoples Republic of Bangladesh, the prevalence of ASD is 0.15% (Global Autism Movement & Bangladesh, 2014). A prevalence of 3% in Dhaka city and 0.068% in rural areas was reported (Non-Communicable Diseases Control [NCDC] et al., 2013). Two early studies using a community-level approach found a prevalence of ASD of 0.02% and 0.08% (Mullick & Goodman, 2005; Rabbani et al., 2009). Recently, another study conducted in a rural community in Bangladesh, reported a prevalence of 0.075% (Akhter et al., 2018).

The increase in prevalence over the years has intensified the demand for practices that work for children with ASD. As a consequence, a wide array of treatments has been developed and various types of effective treatment are available in most high-income countries (HIC; National Autism Center, 2015; National Research Council, 2001; Odom et al., 2010, 2012; Wong et al., 2014, 2015). The National Standards Project (National Autism Center, 2009, 2015) and the National Professional Development Center on Autism Spectrum Disorder conducted comprehensive and systematic reviews of the literature and identified 27 EBPs (National Professional Development Center on Autism Spectrum Disorder [NPDC], 2017a; 2017b). Recently, the National Clearinghouse on Autism Evidence and Practice (NCAEP) identified 28 effective EBPs (Steinbrenner et al., 2020).

There are substantial differences in the financing for the treatment and prevention of neurodevelopmental disorders (NDD) across countries. The lifetime societal cost of supporting an individual with ASD in a HIC was $2.4 million per individual in the United States and £1.5 m in the UK, of which 56% is accounted for by service use (Buescher et al., 2014; Iemmi et al., 2017; Olusanya et al., 2018). Children and adolescents with ASD also exhibit higher rates of healthcare utilization and higher medical care costs compared to other NDDs (Croen et al., 2006; Kim et al., 2020; Tregnago & Cheak-Zamora, 2012). Financing for the treatment and prevention of mental disorders remains insufficient in LIC and LMIC. Median annual mental health expenditures per capita range from US$ 0.20 in LIC to US$ 44.84 in HIC (World Health Organization, 2011). As a consequence, there often is a shortage in mental health care provision, an absence of government initiatives, insufficient training, and a lack of research in LIC and LMIC (Adugna et al., 2020; Blake et al., 2017; Dababnah & Bulson, 2015; Eid et al., 2017; Harrison et al., 2016; Saraceno et al., 2007; Saxena et al., 2007).

## Health Care System and ASD Support in Bangladesh

In this paper, we focus on the implementation of EBPs for autism care in Bangladesh. Bangladesh is one of the most densely populated countries in the world. The majority of its population continues to live by subsistence farming in rural areas. Over the last few decades, Bangladesh has made remarkable progress in poverty reduction, supported by sustained economic growth. As a result, Bangladesh joined the LMIC category on 1 July 2015 (The World Bank, 2020).

Despite the current economic growth and the constitutional obligation of the government to provide health care services to all citizens, health and education levels are relatively poor. Basic health services are provided by four key actors: government institutions, private sector, nongovernmental organizations (NGOs), and donor agencies (Bangladesh Health System Review, 2015). Health staff and specialists are mostly concentrated in urban areas and are much more likely to provide services to upper socioeconomic status (SES) families (Ahmed et al., 2015), whereas the people of rural areas have little access to facilities (Ahmed et al., 2011). Access to specialty mental health care is limited due to missing resources, few skilled professionals, and the concentration of resources in the capital and a few urban areas. Mental health expenditures from the Ministry of Health and Family Welfare amount to less than 0.5% of the total expenditure on health (Alam et al., 2020; Hasan et al., 2021).

Public healthcare for children with disabilities is coordinated by the Ministry of Health and Family Welfare (MOHFW), which has made considerable efforts to strengthen the public sector health system. Specialist services for children with disabilities including autism are managed by the respective public institutes (e.g., the National Institute of Mental Health, the Institute of Pediatric Neurodisorders and Autism).

Private sector organizations and NGOs have played a key role in the promotion of human rights and in the provision of support services for children with disabilities. They cooperate with the government in developing policies and programs. A number of disability service and support centers are functioning in the capital city Dhaka (e.g., The Autism Welfare Foundation, The Rainbow Autism Care and Therapy Center).

Approximately 100 registered schools in Bangladesh address the special needs of children with disabilities (Ehsan et al., 2018). Most of the schools are run by private organizations and NGOs. Others are operated by the Jatiyo Pratibondhi Unnayan Foundation (JPUF), under the Ministry of Social Welfare (e.g., Society for the Welfare of Autistic Children (SWAC) School for Autism, Beautiful Mind).

In the last ten years, the Government of Bangladesh (GOB) has taken several steps toward providing essential services to people with NDD. Two important legal acts have been enacted: (i) The Persons with Disabilities’ Rights and Protection Act 2013 and (ii) The Neuro-Developmental Disability Protection Trust Act 2013. In 2012, a National Steering Committee for Autism and NDD (NSCAND) was established. It focuses on increasing awareness and addressing the situation of children with Autism and NDD. In 2016, the “Situation Assessment of Autism and Neurodevelopmental Disorders in Bangladesh” was published. It found that targeted government-implemented strategies and interventions are required to reduce the socioeconomic, health, and educational disparities impacting those with NDD and their families. The report recommended a phased implementation of a national strategic plan for neurodevelopmental disorders from 2016 to 2021, with the objective to ensure a better quality of life for children with autism and other NDDs (National Strategic Plan for Neurodevelopmental Disorders 2016). Apart from measures to increase public awareness of ASD and NDD, their early identification, early intervention, and employment services, the more widespread use of EBPs was considered a crucial aspect to improve the care provided to patients and their families.

## Attitudes Towards EBP^1^ and EBPs^1^

Many of the organizations and institutions described in the previous section strive to implement EBPs (e.g., applied behavior analysis, social skill training, parent-mediated interventions) to improve the care provided to children and adolescents with autism and other NDDs. There are many factors that may influence the adoption of EBP as a general approach to clinical work and the usage of specific EBPs (cf. Michie et al., 2005). Among them are both organizational-level as well as individual-level factors. A recent overview of the major theoretical frameworks (Williams & Beidas, 2019) identified ten individual-level factors shared by the frameworks. Among them are the attitudes of the professionals, which together with knowledge, skills, self-efficacy expectations, and social norms affect professionals’ intentions and usage of EBPs. The same review also identified seven organizational level factors, including leadership, implementation climate, and available resources. The present research is guided by the findings of this review of theories.

Attitudes are an important individual-level factor and are a determinant of the decision of whether to try and later implement a new EBP (Aarons et al., 2012; Burgess et al., 2017; Frambach & Schillewaert, 2002; Glasman & Albarracin, 2006). For example, Aarons (2004) showed that therapists who had a negative attitude towards using EBPs were significantly less likely to use them in practice. Therefore, it is important to measure professionals’ attitudes towards EBP for children and adolescents with ASD. In Bangladesh, these professionals include mental health professionals as well as specialized teachers. Hence, the first goal of the present study was to examine the attitudes towards EBP of professionals who provide care to children with ASD. As there are many different types of EBPs, we intended to study professionals’ general attitudes towards EBP and their usage of specific forms of treatments being considered for wider implementation in Bangladesh. The findings will show whether mental health care providers have attitudes which support the implementation and use of EBPs.

Research also showed that attitudes towards EBP may differ as a function of demographic variables and organizational factors (Aarons, 2004, Aarons et al., 2010, 2011, 2012; Beidas et al., 2015; Connors et al., 2019; Damschroder et al., 2009; Greenhalgh et al., 2004; Locke et al., 2019; Okamura et al., 2018; Powell et al., 2017; Rye et al., 2019; Smith, 2013; Vassos et al., 2016; Wisdom et al., 2014). For instance, females, compared to males, have reported a greater perceived fit of EBPs with the characteristics and needs of patients (Aarons et al., 2010, 2012; Rye et al., 2019). Younger participants considered EBP more relevant for job security and observed more organizational support for learning new EBPs (Aarons et al., 2009; Okamura et al., 2018; Rye et al., 2019). Providers with higher caseloads have perceived greater administrative burdens related to the use of EBPs and clinicians working in public clinical settings, compared to private clinical settings, have reported a poorer fit of EBP with their clinical practice (Aarons et al., 2012). Higher levels of experience were associated with a perception of therapy as a balance between art and science (Aarons et al., 2012). Less experienced clinicians claimed a greater openness to trying new interventions and a higher willingness to try or use more structured or manualized interventions (Aarons et al., 2010). Therefore, the present study explored how providers’ demographic variables are related to attitudes to and adoption of EBPs in Bangladesh.

## Barriers and Facilitators to the Implementation and Usage of EBP and EBPs

Research also showed that the adoption and successful implementation of EBPs depend on professional, social, organizational, and contextual factors (Aarons, 2004, 2005; Glisson & Schoenwald, 2005; Glisson et al., 2008; Greenhalgh et al., 2004; Grol & Wensing, 2004; Lau et al., 2015; Raghaven et al., 2008). In their systematic review of reviews, Lau et al. (2015) identified a list of crucial organizational and contextual barriers, for example, support and guidance by superiors, and available resources including time.

As no research on this topic has been conducted in Bangladesh, the second objective of our study was to assess, which of the potential organizational and contextual barriers to the use of EBPs for ASD are present in Bangladesh. From the factors identified by Lau and colleagues we selected variables which are potentially relevant in Bangladesh (cf. Khan et al., 2018; Mannan, 2017, see method section for details).

## Research Questions and Hypotheses

**Fig. 1:**
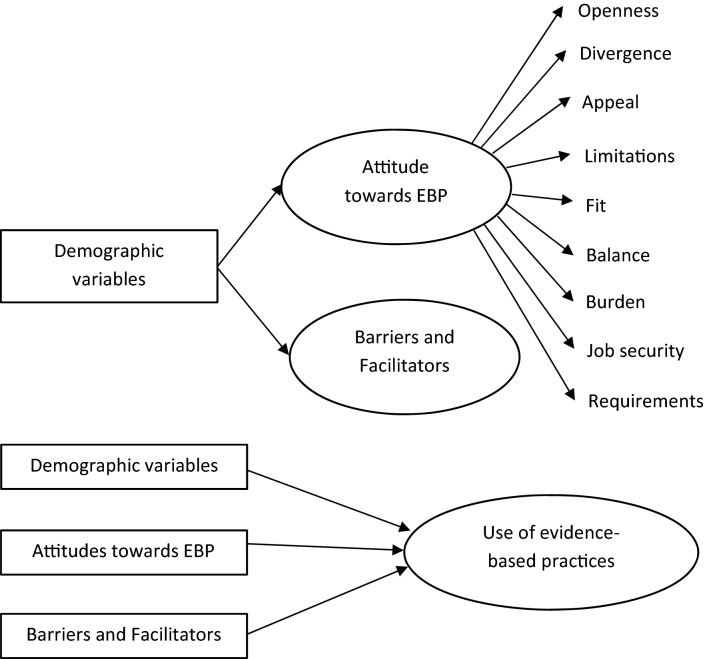
Conceptual framework of the present study. Predictors are shown by squared boxes, predicted outcomes by circles. In the upper half the investigated relations between demographic variables as predictors and attitudes as well as barriers as outcome variables are shown. In the lower half, the relation between demographic variables, attitude, and barriers as predictors and the use of EBPs as outcome are shown

The conceptual framework of the study is illustrated in Fig. 1. The framework builds upon theoretical models of EBP implementation including the Consolidated Framework for Implementation Research (CFIR; Damschroder et al., 2009), the theoretical review by Williams and Beidas (2019), and the empirical research summarized in the previous section. We decided not to use a specific theoretical framework from the literature, as these tend to be more comprehensive and we were not able to cover all aspects due to limited available resources. Instead, we focused on professionals’ attitudes on one hand and potential barriers on the other hand.

Based on the conceptual framework shown in Fig. 1, the following research questions were addressed:

- What are the attitudes of mental health professionals and special teachers in Bangladesh towards EBP for children and adolescents with ASD?
- What are the potential barriers to the use of EBPs perceived by these professionals?
- Do demographic variables predict the attitude towards EBP?
- Which EBPs^2^ for children and adolescents with ASD do mental health professionals and special teachers in Bangladesh use?
- Do attitudes towards EBP, perceived barriers, and demographic variables predict the number of different EBPs that are being used?

Based on the conceptual framework and the previous literature (Aarons, 2004; Aarons et al., 2010, 2012; Lau et al., 2015), hypotheses were developed prior to data collection and examined in this study. We anticipated that attitudes toward EBP (Hypothesis 1), perceived barriers for implementing and using EBPs (Hypothesis 2), and the types of interventions being used (Hypothesis 3) would vary by professionals’ demographic characteristics and the settings they worked in. However, given the lack of prior literature conducted in South Asia, the analyses with respect to these hypotheses were exploratory in nature. As visualized in the conceptual framework, we hypothesized that more positive attitudes and fewer perceived barriers would be related to using a broader variety of EBPs (Hypothesis 4).

Research questions were addressed and hypotheses were tested by conducting a survey among professionals working with children and adolescents with ASD in a clinical or a school setting.

## Method

### Settings and Participants

We conducted a cross-sectional survey (Lavrakas, 2008) among professionals in the Urban area of Dhaka, who provide services for children and adolescents with ASD. We recruited participants from three service settings (public clinical, private clinical, and special school). Public organizations provide comprehensive services to individuals with NDD, including autism. Private and non-governmental autism centers and clinics offer a range of services to children with ASD. Special schools provide care for children with autism and other disabilities, who cannot attend mainstream educational facilities. Typically, the schools are providing speech and language therapy, sensory integration therapy, behavior therapy, music therapy, play therapy, occupational therapy, and conventional educational services. A list of potentially eligible professionals was collected from the websites of the Bangladesh Association of Psychiatrists, Bangladesh Society for Child Neurology, Bangladesh Clinical Psychology Society, Bangladesh School Psychology Society, Bangladesh Psychological Association, Disability service, and support centers, Institute of Special Education and Special Schools.

Inclusion criteria were the following: Mental health professionals (psychiatrists, pediatric neurologists, psychologists, developmental psychologists, counselors) and/or special teachers working with children and adolescents with ASD and their supervisors. Participants had to be fluent in Bangla or English. Exclusion criteria were: (i) Professionals not working with children and adolescents with autism and (ii) Professionals in training.

Concerning sample size, we strived for a sample as large as possible to get representative results for research questions 1, 2, and 4. A sample size calculation with respect to the hypotheses concerning the prediction of attitudes towards EBP and perceived barriers yielded a target sample size of N = 157, assuming a medium effect of a model with seven demographic variables as predictors, a significance level of 0.05, an intended power of 0.8, and ten models to be tested.

### Measures

The survey comprised of multiple-choice items and open-ended questions. Whenever possible, scales and items of existing questionnaires in English were used. The survey was available in English and Bangla. A main part of the survey consisted of the nine subscales of the Evidence-based Practice Attitude Scale-36 (EBPAS-36; Rye et al., 2017, see below for details). The EBPAS has been used in culturally diverse contexts. It has been found to have sound content validity and internal consistency. A translation and back-translation procedure for all items was used to create the Bangla version of the questionnaire. Translators were academic researchers familiar with ASD and the study of professionals’ attitudes (the first and second author of the paper). The process included several rounds of translation and back-translation with rigorous comparisons between the original and the translated versions according to guidelines for questionnaire adaptation in order to achieve the highest possible content validity. The dimension of EBPAS-36 that was most challenging to translate was the divergence subscale. A pre-test with mental health clinicians working at the University of Dhaka was conducted to ensure that all items were understood correctly. Due to limited resources, a separate evaluation of the questionnaire and its psychometric properties was not possible.

#### Demographic Variables

These included gender, age, years of experience (the number of years working as a mental health professional), caseload per year (the number of patients worked on average per year), workplace (public, private, special school), professional background (medicine, psychiatry, psychology/clinical psychology, counseling, special teachers and others), and theoretical orientation (psychoanalytic/psychodynamic, cognitive-behavioral, behavioral, humanistic, eclectic and others).

#### Attitudes Toward EBP

Attitudes toward EBP were measured using the EBPAS-36 (Rye et al., 2017, a short version of the Evidence-based Practice Attitude Scale-50 (EBPAS-50; Aarons et al., 2012). It assesses mental health providers’ attitudes towards EBP including the usage of EBPs. It was validated with US and Norwegian samples (Aarons et al., 2012; Rye et al., 2017), but not in the context of Bangladesh. According to our inclusion criteria, all our participants were academically trained and many had spent time abroad during their studies. Therefore, we considered it adequate to use a Western validated instrument. As described above, the questionnaire was translated into the local language. Participants rated their agreement with the presented statements using a five-point Likert scale (from 0 “not at all” to 4 “to a very great extent”). The EBPAS-36 contains 12 subscales, each with 3 items, and a total score indicating attitudes towards EBP in general. For the present study, nine subscales were used, which we considered appropriate for the context of Bangladesh: *openness* to using EBPs (e.g., ‘I like to use new types of therapy/interventions to help my clients’), the perceived *divergence* of professionals’ usual practice from evidence-based interventions (e.g., ‘Clinical experience is more important than using manualized therapy/treatment’), the intuitive *appeal* of adopting EBPs (e.g., ‘If you received training in a therapy or intervention that was new to you, how likely would you be to adopt it if it made sense to you?’), *limitations* of EBPs and their inability to address client needs (e.g., ‘EBP is not useful for clients with multiple problems’), EBPs’ *fit* with the values and needs of the client and therapist (‘If you received training in a therapy or intervention that was new to you, how likely would you be to adopt it if it fit with your clinical approach?’), *balance* between perceptions of clinical skills and science as important in service provision (e.g., ‘Therapy is both an art and a science’), the time and administrative *burden* with learning EBPs (e.g., ‘I don’t have time to learn anything new’), *job security* related to expertise in EBPs or professional marketability provided by learning an EBP (e.g., ‘Learning an EBP will help me keep my job’), and the likelihood of adopting EBPs given *requirements* to do so (e.g., ‘If you received training in a therapy or intervention that was new to you, how likely would you be to adopt it if it was required by your regulatory body?’). Rye et al. (2017) confirmed the factor structure of the EBPAS-36. Subscales had good internal consistency.

#### Barriers and Facilitators

Organizational and contextual barriers to implementing EBPs, which were identified in the systematic review of reviews by Lau et al. (2015), were assessed using 13 newly constructed items. Items were formulated as statements (e.g., “My organization’s goals and objectives support the usage of EBPs”; “The current policy and legislative framework in Bangladesh enable me to use EBPs”; see Fig. 3 for all items). Participants indicated their agreement with the statement on a five-point Likert scale ranging from 0 (“Not at All”) to 4 (“To a Very Great Extent”). A higher total score indicates that more facilitators and lower score that more barriers are perceived. Given that the items were based on existing findings (Lau et al., 2015), the content validity related to barriers and facilitators to implementing EBPs can be considered good.

#### Current Use of EBPs

Use of nine types of EBPs that were found to be effective by the National Standard Project (NSP) and the National Professional Development Center (NDPC) (Wong et al., 2014, 2015) and the systematic review of medical treatments for children with ASD (McPheeters et al., 2011) was examined. The EBPs were: Behavioral Interventions (e.g. imitation training, reinforcement schedules, video modeling), Cognitive Behavioral Intervention (i.e., behavior modification and cognitive restructuring), Comprehensive Behavioral Treatment for Young Children (e.g. the UCLA Young Autism program by Lovaas and colleagues), Language Training (e.g. Functional verbal communication), Naturalistic Teaching Strategies (e.g. verbal prompting), Parent Training (i.e., individual or group training by using training manuals), Peer Training (e.g., peer networks, integrated playgroups), Social Skills Training (i.e., behavioral interventions to improve social and communicative competencies), and Antipsychotic Medications (Risperidone, Aripiprazole). The examples given here were also provided in the survey. Participants were asked to indicate whether they currently use the type of treatment by ticking a check box.

### Ethical Considerations

The present study was approved by the Ethics Committee of Bangladesh Medical Research Council (BMRC; Ref: BMRC/NREC/2019-2022/386). Participants were informed that the survey was anonymous and the responses of the participants would be stored electronically on secured servers at the University of Goettingen, Germany, and the University of Dhaka, Bangladesh, to ensure confidentiality. At the end of the survey, contact details of the participants were removed from all records.

### Procedure

Initially, eligible professionals in Bangladesh were contacted by phone, email, or met in person to make an appointment for the paper survey. After getting a positive response, research assistants met the participant in person, distributed an information sheet, which described the purpose of the study and the rights of the participants. All participants were assured that participation was voluntary and that they could stop the survey at any time without giving reasons. When participants agreed to participate, they signed the consent form and then filled in the questionnaire. Filling in the questionnaire took on average 45 min. Participants returned the completed surveys to the research assistants. The research assistants were present to give instructional support and to thank participants for completing the survey.

### Statistical Analyses

All statistical analyses were performed using R (R Core Team, 2020). *P*-values less than 0.05 were considered statistically significant. Descriptive statistics were computed for all variables of interest. Means and standard deviations for the scores of the EBPAS-36 subscales as well as all barriers and facilitators items were calculated. Internal consistencies of subscales were assessed by Cronbach’s alpha. Types of EBPs used were described by frequency rates.

To test Hypothesis 1, general linear models were built to assess the relationships between attitudes towards EBP (EBPAS-36 subscales) and demographic variables of Bangladeshi mental health professionals (working experience, caseload per year, gender, age, and service setting). Note that respondents’ professional background and theoretical orientation were not included in the analyses due to multicollinearity with other predictors. Separate models were created for the scores of the nine subscales. To correct for multiple testing, a significance level of alpha = 0.05/9 = 0.0056 was used. For each significant model, ANOVAs were computed for the predictor variables. Again, we corrected for multiple testing.

To test Hypothesis 2, a general linear model for the sum score of the barriers and facilitators items and the demographic variables as predictors was set up and tested for significance. To find out which demographic variables (working experience, caseload per year, gender, age, and service setting) predict the perceived barriers, ANOVAs were run. Again, a correction for multiple testing was used.

To test Hypothesis 3, model comparisons were computed for each of the nine practices inquired about. A model including the factor service setting as a predictor of the usage of the respective type of intervention was compared to a model without the predictor. Due to the outcome being a categorical variable (usage yes *vs.* no) a likelihood ratio test was used for statistical comparisons. To correct for multiple testing, a significance level of alpha = 0.05/9 = 0.0056 was used.

To test Hypothesis 4, a general linear model was used to model the relationship between attitudes towards EBP, demographic variables, and perceived barriers and the number of different types of EBPs used. ANOVAs were run to assess the significance of individual predictors.

## Results

### Participants

**Fig. 2:**
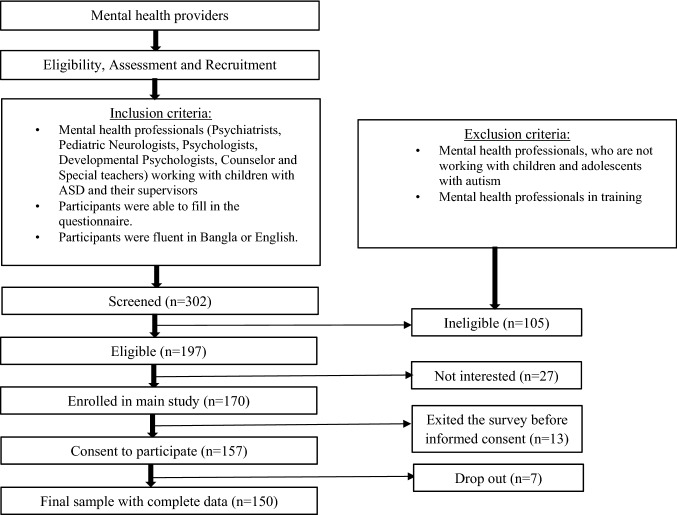
Flow of participants

Three hundred and two potential participants were identified through the initial search. Of them, 105 did not meet the inclusion criteria and were excluded. The total number of eligible participants was 197, of which 27 were not interested to participate. During this stage, one hundred and seventy participants were enrolled. Of the 170 participants, 13 participants declined to participate in the survey before signing the consent form. Written consent was obtained from 157 participants. Participants worked in seven public and seven private organizations serving children with ASD and seven special schools. Seven participants refused to complete the survey. A total of 150 mental health professionals and special teachers completed the survey (see Fig. 2 for the flow of participants). Thus, the response rate was 76.1%.

The mean age was 30.5 years and the majority of the participants fell into the age category of 21 to 30 years (n = 86, 57.33%). There were 107 females (71%) and 43 males (29%), who completed the survey. Professionals’ years of experience ranged from two months to twenty-five years (Mean = 4.93 years, SD = 4.60). Participants had an average caseload of 416, but numbers varied widely. Therefore, caseload was classified into three categories (see Table 1). In terms of service settings, 47.33% of participants worked in private clinical settings, 22% in public clinical settings, 27.33% in school settings; the remaining 3.34% were missing. Professions were special teachers (27.33%), medicine (11.33%), counselling (8.67%), psychology (12.67%). The majority of respondents reported having a variety of different professional backgrounds (n = 58, 38.67%). These professionals were assigned to the “other” category. Only 2 respondents (1.33%) did not report any profession. The respondents’ theoretical orientations were eclectic (44%), cognitive-behavioral (36.67%), and others (18.66%), with data missing for one respondent. Participant demographic characteristics are provided in Table 1.

**Table 1:** Demographic characteristics of participants (N = 150)

Characteristics	Mean	SD
Age	30.5	7.79
Years of experience	4.93	4.60
Caseload (per year)	416.30	863.36

	N	%
*Gender*		
Female	107	71.33
Male	43	28.67
*Service settings*		
Private	71	47.33
Public	33	22.00
Special school	41	27.33
Missing	5	3.34
*Professional background*		
Medicine	17	11.33
Psychology	19	12.67
Counselling	13	8.67
Special teachers	41	27.33
Other	58	38.67
Missing	2	1.33
*Theoretical background*^a^		
Cognitive-behavioral	55	36.67
Eclectic	66	44.00
Other	28	18.66
Missing	1	0.67

^a^Theoretical background refers to the theoretical framework of the intervention models the professionals are using

### Attitudes Towards EBP

First, the internal consistencies of the nine EBPAS-36 subscales used in this study were analyzed. Six subscales had acceptable to excellent internal consistencies: Openness, Limitations, Appeal, Fit, Job security, and Requirements (see Table 2 for details). The Divergence, Burden, and Balance subscales, however, had low consistencies (0.28, 0.48, and 0.56), which did not improve removing the least consistent item (Gliem & Gliem, 2003).

**Table 2:** Attitudes towards evidence-based practice, mean ratings EBPAS-36 subscales, standard deviations, confidence intervals (CI), and Cronbach’s alpha values of internal consistency

Sub-scales	*M*	*SD*	*CI*	Cronbach's *α*
Openness	2.94	0.82	[2.81, 3.07]	0.75
Divergence	2.33	0.80	[2.20, 2.46]	0.28
Appeal	3.06	0.79	[2.93, 3.19]	0.69
Limitations	2.51	1.03	[2.35, 2.67]	0.60
Fit	2.87	0.87	[2.73, 3.01]	0.76
Balance	1.26	0.83	[1.13, 1.39]	0.56
Burden	2.90	0.85	[2.76, 3.04]	0.48
Job-security	3.29	0.83	[3.16, 3.42]	0.86
Requirements	3.14	1.05	[2.97, 3.31]	0.87

Scores were inverted for the subscales divergence, limitations, balance, and burden as recommended by the manual. Scales range from 0 “not at all” to 4 “to a very great extent”

Mean ratings (and SD) of EBPAS-36 subscales can be found in Table 2. Overall, Bangladeshi mental health professionals had a mean score of 2.70 on the nine subscales, which indicates an overall favorable attitude towards EBP. Most participants assumed that learning an EBP and having respective expertise increases their job prospects (job security subscale). They also would be likely to adopt EBPs if it was required by the supervisor, agency, or state (requirements subscale). They also claimed that they would be likely to adopt an EBP if it ‘made sense’ to them, could be used correctly, or was being used by colleagues, who were happy with it (appeal subscale). Most were open to trying new interventions and would be willing to use structured or manualized interventions. Many professionals perceived a fit of EBP with the characteristics and needs of the client and clinician. Some participants, however, perceived an inability of EBPs to address client needs (limitations subscale). The mean scores of the divergence, balance, and burden subscales were difficult to interpret due to their low internal consistency.

### Perceived Barriers

The Cronbach alpha value of the thirteen items of the barrier and facilitators subscale was α = 0.88, which indicates a high level of internal consistency. The mean score of all items was (2.41 ± 0.73), indicating that many participants perceived conditions as facilitating the widespread use of EBPs for ASD.

**Fig. 3:**
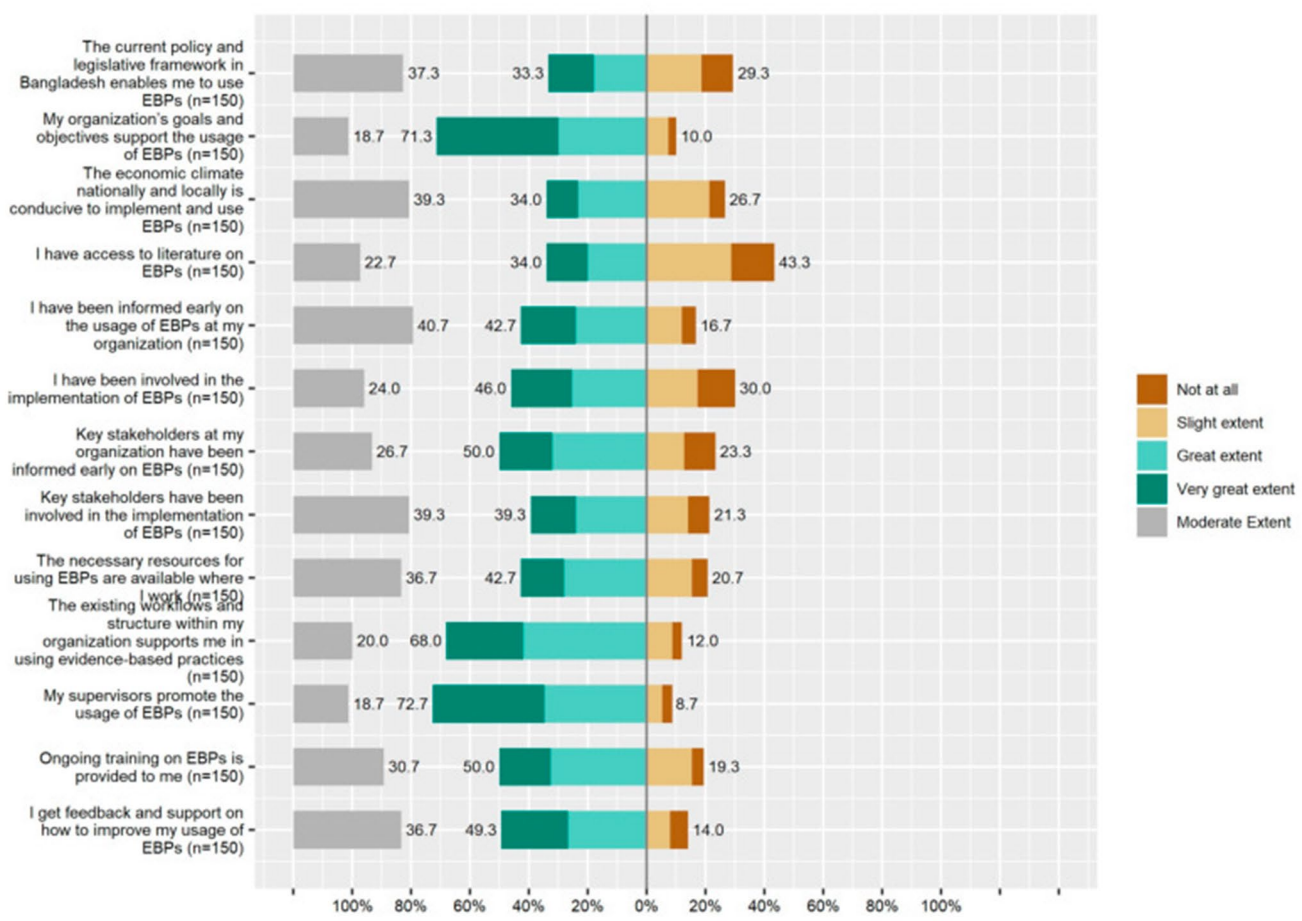
Perceived barriers

To simplify the results regarding individual barriers, responses were categorized as disagree (i.e., not at all or slight extent) or agree (i.e., great extent or very great extent), while the response “I agree to some extent” was considered neutral. Figure 3 shows these results. A majority of professionals (72.7%) agreed that their supervisors promoted the usage of EBPs compared to only 8.7% of practitioners, who disagreed. In addition, the majority of the professionals (71.3%) agreed that their organizations’ goals and objectives were supportive to use the EBPs and that the existing workflows and structures within their organizations were helpful to use EBPs (68%). On the other hand, 43.3% disagreed with the statement “I have access to literature on EBPs (e.g. clinical or academic journals)”, whereas 34% said that they had access. Half of the participants said that ongoing training on EBPs was provided to them, but 19.3% said that an ongoing training program was not available. Almost half of the participants (49.3%) agreed that they received feedback and support from their organizations to learn the usage of EBPs. Forty-six percent of participants said they were involved in the implementation of EBPs. Thirty-four percent agreed with the statement “The economic climate nationally and locally is conducive to implement and use EBPs” whereas 26.7% disagreed. Thirty-three percent agreed with the item “The current policy and legislative framework in Bangladesh enable me to use EBPs”, while 29.3% did not. Forty-three percent agreed that the necessary resources (e.g. funding, adequate staff with appropriate skills, training, and ongoing support) for using EBPs were available where they worked, but 20.7% claimed they were not (see Fig. 3 for more results).

### Relationships Between Attitudes Towards Evidence-Based Practice, Perceived Barriers, and Demographic Variables

Results are shown in Table 3. Results are provided for all EBPAS-subscales although three had low internal consistencies (divergence, balance, burden). Some demographic variables (gender, age, working experience, caseload per year, and service settings) predicted some types of attitudes towards EBP. For five of the nine subscales, no demographic predictors were significant (openness, divergence, limitations, burden, and job security, see Table 3 for details). Models were significant for the appeal, fit, balance, and requirements subscales and explained between 20% (balance) and 34% (fit) of the variance. Patients seen per year and the service setting were the most consistent predictors of attitudes. For the appeal subscale, caseloads per year (*F* (2,129) = 6.863, *p* = 0.001) and service setting (*F* (2,129) = 9.645, *p* = 0.001) were significant predictors. For perceived fit, working experience (*F* (1,120) = 13.162, *p* = 0.001), caseload per year (*F* (2,120) = 14.943, *p* = 0.001), and service setting (*F* (2,120) = 7.782, *p* = 0.001) were significant predictors. For balance (i.e., the matching of EBP with client´s and clinician´s values and needs), caseload per year (*F* (2,122) = 7.916, *p* = 0.001) and service setting (*F* (2,122) = 4.840, *p* = 0.01) were significant predictors. Requirement subscale scores were significantly predicted by caseload per year (*F* (2,128) = 6.011, *p* = 0.01), and service setting (*F* (2,128) = 9.053, *p* = 0.001). The mean ratings of the barriers and facilitators scale were not significantly related to professionals’ demographic variables.

**Table 3:** Relationships between attitudes towards evidence-based practice (EBPAS-36 subscales), perceived barriers, and demographic variables of Bangladeshi mental health professionals

Outcomes EBPAS Subscales and Perceived Barriers	Predictors	*df*	*Mean square*	*F*	*p*-value	Model test
Openness	Gender	1	0.041	0.061	0.805	*R*^*2*^ = 0.075 *F* (7,128) = 1.475
	Age	1	0.146	0.217	0.642	
	Working experience	1	0.006	0.008	0.927	
	Caseload (per year)	2	2.165	3.214	0.043	
	Service settings	2	1.218	1.807	0.168	
Divergence	Gender	1	3.263	5.447	0.021	*R*^*2*^ = 0.118 *F* (7,121) = 2.320
	Age	1	0.059	0.099	0.754	
	Working experience	1	0.029	0.048	0.828	
	Caseload (per year)	2	1.163	1.942	0.148	
	Service settings	2	2.026	3.383	0.040	
Appeal	Gender	1	0.001	0.002	0.989	*R*^*2*^ = 0.216 *F* (7,129) = 5.086*
	Age	1	1.127	2.194	0.141	
	Working experience	1	0.202	0.392	0.532	
	Caseload (per year)	2	3.526	6.863	0.010*	
	Service settings	2	4.955	9.645	0.001*	
Limitations	Gender	1	0.451	0.402	0.527	*R*^*2*^ = 0.024 *F* (7,128) = 0.452
	Age	1	0.017	0.015	0.904	
	Working experience	1	1.186	1.058	0.306	
	Caseload (per year)	2	0.233	0.208	0.813	
	Service settings	2	0.714	0.637	0.531	
Fit	Gender	1	0.268	0.532	0.467	*R*^*2*^ = 0.335 *F* (7,120) = 8.640 *
	Age	1	0.671	1.333	0.251	
	Working experience	1	6.621	13.162	0.001*	
	Caseload (per year)	2	7.517	14.943	0.001*	
	Service settings	2	3.915	7.782	0.001*	
Balance	Gender	1	0.587	1.002	0.319	*R*^*2*^ = 0.197 *F* (7,122) = 4.264*
	Age	1	1.633	2.784	0.098	
	Working experience	1	0.322	0.549	0.460	
	Caseload (per year)	2	4.642	7.916	0.001*	
	Service settings	2	2.838	4.840	0.010*	
Burden	Gender	1	1.069	1.449	0.231	*R*^*2*^ = 0.069 *F* (7,125) = 1.316
	Age	1	1.388	1.880	0.173	
	Working experience	1	0.068	0.093	0.761	
	Caseload (per year)	2	2.045	2.770	0.067	
	Service settings	2	0.093	0.126	0.881	
Job security	Gender	1	0.225	0.320	0.573	*R*^*2*^ = 0.039 *F* (7,127) = 0.734
	Age	1	0.003	0.004	0.949	
	Working experience	1	0.278	0.394	0.531	
	Caseload (per year)	2	0.106	0.150	0.861	
	Service settings	2	1.453	2.059	0.132	
Requirements	Gender	1	0.067	0.081	0.777	*R*^*2*^ = 0.219 *F* (7,128) = 5.130*
	Age	1	3.369	4.082	0.045	
	Working experience	1	1.334	1.617	0.206	
	Caseload (per year)	2	4.961	6.011	0.003*	
	Service settings	2	7.473	9.053	0.001*	
Barriers and facilitators	Gender	1	0.428	0.780	0.380	*R*^*2*^ = 0.067 *F* (7,113) = 1.155
	Age	1	0.935	1.702	0.195	
	Working experience	1	0.001	0.001	0.987	
	Caseload (per year)	2	1.228	2.237	0.112	
	Service settings	2	0.310	0.564	0.570	

*p-value < 0.05 after correcting for multiple testing

### EBPs Currently Used by Mental Health Professionals Who are Working in Public, Private, and Special School Settings

Sixteen percent of respondents (n = 24) did not use any of the nine types of interventions, 12.7% (n = 19) of participants reported that they were using one type of intervention, 2.7%, (n = 4) selected two, 12.7% (n = 19) three, 10.7% (n = 16) four, 14.0% (n = 21) five, 10.7% (n = 16) six, 11.3% (n = 17) seven, and 9.3% (n = 14) reported using eight different types of interventions.

Table 4 shows the types of interventions being used by professionals as a function of service setting. As expected, some differences between service settings were found. The use of social skills training, cognitive-behavioral intervention, and language training were reported by more than 80% of the respondents in special school settings, while less than half of the respondents from private and public clinical settings reported using them. We found significant differences among service settings concerning the usage of all different interventions other than parent training interventions (see Table 4 for details). Providers in special school settings were more likely than those in public and private clinical settings to use social skills training, cognitive-behavioral intervention, and language training. In addition, more than 75% of the respondents from special schools indicated that they were currently using behavioral interventions and naturalistic teaching strategies, 63.4% were implementing parent training, and 53.7% were using peer training. These types of interventions were reported considerably less often in public and private clinical settings. Overall, comprehensive behavioral treatments and antipsychotic medications were used by less than 1 in 4 respondents (see Table 4 for details).

**Table 4:** EBPs currently used by mental health professionals, who are working with children and adolescents with ASD in different service settings

Intervention type	Private clinical setting		Public clinical setting		Special school setting		Model test for service setting
	n	%	n	%	n	%	
Behavioral Interventions (e.g., imitation training, reinforcement schedules, video modeling)	29	40.9	16	48.5	31	75.6	*Chi*^2^(2) = 13.37*
Cognitive Behavioral Intervention(i.e., behavior modification and cognitive restructuring)	31	43.7	10	30.3	35	85.4	*Chi*^2^(2) = 28.77*
Comprehensive Behavioral Treatment for Young Children (e.g., LOVAAS program)	14	19.7	5	15.2	19	46.3	*Chi*^2^(2) = 11.62*
Language Training(e.g., Functional verbal communication)	29	40.9	17	51.5	33	80.5	*Chi*^2^(2) = 17.62*
Naturalistic Teaching Strategies (e.g., verbal prompting)	22	31.0	17	51.5	32	78.1	*Chi*^2^(2) = 24.18*
Parent Training (i.e., individual or group training by using training manuals)	35	49.3	12	36.4	26	63.4	*Chi*^2^(2) = 5.48
Peer Training (e.g., Project LEAP, peer networks, integrated play groups)	15	21.1	9	27.3	22	53.7	*Chi*^2^(2) = 12.67*
Social Skill Training (i.e., behavioral interventions to improve social and communicative competencies)	36	50.7	15	45.5	35	85.4	*Chi*^2^(2) = 17.93*
Antipsychotic Medications (Aripiprazole, Risperidone, Chlorpromazine, Prochlorperazine, Clozapine)	17	23.9	5	15.2	0	0	*Chi*^2^(2) = 17.22*

*p-value < 0.05 after correcting for multiple testing

### Relationships Between the Number of EBPs Used, Attitudes to EBP, and Demographic Variables

Professionals’ attitudes towards EBP, perceived barriers, and the five demographic variables (age, gender, working experience, caseload per year, and service settings) were included as predictors in a model with the number of EBPs used as the outcome variable. The results are presented in Table 5. The overall model was significant *F* (17, 73) = 2.908, *p* = 0.001) and explained about 40% of the variance in the number of different practices used. ANOVAs revealed that only service setting (*F* (2, 73) = 9.926, *p* = 0.001) and the requirement subscale were significant predictors (*F* (1, 73) = 24.448, *p* = 0.001). The result indicates that professionals used a broader variety of EBPs when they agreed that they would adopt an EBP if it was required by the supervisor, agency, or state. As shown in Table 4, special school teachers used more EBPs than professionals in other settings.

**Table 5:** Relationships between number of EBPs used, attitudes towards EBP, and demographic variables

Outcome	Predictors	*df*	*Mean square*	*F*	*p*-value	Model test
Number of evidence-based practices used	Gender	1	0.311	0.058	0.810	*R*^*2*^ = 0.404 *F* (17,73) = 2.908*
	Age	1	0.006	0.001	0.973	
	Working experience	1	0.322	0.060	0.806	
	Caseload (per year)	2	1.900	0.356	0.702	
	Service settings	2	52.938	9.926	0.001*	
	Openness	1	0.399	0.075	0.785	
	Divergence	1	0.217	0.041	0.841	
	Appeal	1	0.680	0.128	0.722	
	Limitations	1	0.187	0.035	0.852	
	Fit	1	0.161	0.030	0.863	
	Balance	1	1.870	0.351	0.556	
	Burden	1	6.306	1.182	0.280	
	Job security	1	13.110	2.458	0.121	
	Requirements	1	130.390	24.448	0.001*	
	Barriers and facilitators	1	0.027	0.005	0.944	

*p-value < 0.05 after correcting for multiple testing

## Discussion

The aims of the present study were to examine mental health care professionals’ and special teachers’ attitudes towards EBP and their usage of EBPs for children and adolescents with ASD and to explore how providers’ demographic factors are related to the attitudes and the adoption of EBPs in Bangladesh. A further aim was to identify potential barriers to the implementation and usage of EBPs. Based on the results, recommendations could be derived to support the more widespread use of EBPs.

The findings indicate a generally positive attitude of professionals towards EBP. A majority was open to the usage of manualized EBPs and found them appealing. They agreed that EBPs contribute to job security and that they would use them when required. Such a positive attitude is not unexpected, given the positive value assigned to EBP in academic training programs in Bangladesh and elsewhere. With respect to barriers, we inquired about the external context and the organization the professionals work in. These factors were found to be crucial according to the review of Lau et al. (2015). Many respondents judged the external and the organizational context as facilitating the implementation and usage of EBPs. For example, many agreed that their supervisors, organization, existing workflows and structures support the usage of EBPs.

There was, however, a substantial minority that perceived the situation differently. Roughly 20% said that they did not have the necessary resources and did not receive ongoing training. Given the very limited spending on mental health in Bangladesh (cf. Alam et al., 2020; Hasan et al., 2021), this number is rather low. Note, however, that participants worked in many different institutions, some public, some private and for-profit, and some funded by NGOs.

Participants’ responses were less clear concerning the presence of a national and local economic climate conducive to the implementation and usage of EBPs. Many only partially agreed with the statements on these topics. The same was true for the current policy and legislative framework in Bangladesh. Almost 30% of participants said that the present policies and legislative framework did not enable them to use EBPs. This finding seems surprising given recent legislation and government initiatives (see the introduction for details). One potential explanation might be a lack of awareness of these initiatives. Previous findings in other LMICs indicate that an appropriate legislative mechanism, as well as supportive national and local policies, can promote the implementation of EBPs (Adugna et al., 2020; Fontaine et al., 2010; Leonard et al., 2020). A lack of stated goals, clear priorities, and missing directions were found to be barriers (Eisner et al., 2011). Our findings suggest that such barriers sometimes still exist in Bangladesh.

A considerable number of participants (43%) reported that they had no access to literature on EBPs. This finding, however, is not specific to Bangladesh or other LMICs (see Aarons et al., 2012; Holzer et al., 2007; Kauth et al., 2011; Okamura et al., 2018; Pagoto et al., 2007; Rye et al., 2019; Toth & Manly, 2011).

The first hypothesis was that attitudes towards EBP depend on professionals’ demographic characteristics. This hypothesis was supported by our findings. Clinicians with higher caseloads and clinicians who are working in private clinical and special school settings were more open to EBPs, found them more appealing, judged that they allowed for a good fit of values and needs of patients and clinicians, and said that they were inclined to use them when required. This finding was consistent with some previous findings which indicated that private professionals reported a greater perceived fit of EBPs with the characteristics and needs of clients (Aarons et al., 2012). It was inconsistent with findings by Rye et al. (2019), who found private practitioners to have a lower score on attitudes related to requirements. A possible explanation might be that surveyed clinicians working in a private setting in Bangladesh are less independent than clinicians working in private practice in Western HIC, as many participants worked in institutional settings.

Interestingly, we found no relation between caseload and perceived burden. This was inconsistent with previous findings from HIC that clinicians with higher caseloads associated EBP with more burden (Aarons et al., 2012; Okamura et al., 2018). Maybe the guidance and structure provided by EBPs are considered more helpful than the effort required. Work setting was also not related to the perception of high temporal demands and administrative burden. This finding is consistent with Aarons et al. (2012).

Contradicting to our second hypothesis, we found no relation of demographic characteristics and perceived barriers. This finding was somewhat surprising given the differences between settings in organization, funding, and patients cared for. One reason might be that there is also substantial heterogeneity within service settings when it comes to barriers. A second possible explanation might be that the significant efforts to promote, integrate, coordinate, and expand services to children with ASD have been successful. Another explanation is that organizational initiatives have been undertaken to implement EBPs.

Supporting Hypothesis 3, the number of different EBPs being used varied as a function of service settings. Specifically, professionals who are working in special school settings tended to use these interventions and a broader variety. This could indicate that special teachers seek out a multitude of interventions for children with autism compared to other practitioners. The present study found that social skill training and cognitive-behavioral interventions were the most commonly used practices in special schools and private clinical settings. This might not be surprising given that social-communication skill deficits are a common feature of autism (DSM-5; APA, 2013). Special teachers were also more likely to use peer training programs than clinicians (both in public and private settings). In these programs, peers play an active role in helping children with autism to develop social skills.

The type of treatment used most in all settings was parent-mediated interventions. Utilizing parent-mediated interventions helps to ensure cost-efficiency and sustainability in LMIC (Blake et al., 2017). In addition, non-specialist mediated interventions are particularly important, as preschool-age children with ASD and other NDDs spend most of their waking hours at home with their caregivers (Parsons et al., 2017; Reichow et al., 2013).

Comprehensive behavioral interventions were used by less than 50% of respondents in special school settings and below 20% both in public and private clinical settings. Comprehensive treatment programs are well investigated in HIC (National Research Council, 2001; Odom et al., 2010; Wong et al., 2015), but not in LMIC (Pervin et al., 2021). The high costs of these programs are likely to prevent their application.

Antipsychotic medications were the type of treatment used least. Several reviews have found these medications to be effective for severe behavioral problems at least short term (Ching & Pringsheim, 2012; Fung et al., 2016; Hirsch & Pringsheim, 2016; Khaleghi et al., 2020; McPheeters et al., 2011; Siegel & Beaulieu, 2012; Yu et al., 2020). They were used successfully in LMIC, although few studies have been conducted (Patra & Kar, 2021). Given the considerable side effects, practitioners in Bangladesh may not be confident to use them.

Sixteen percent of respondents used none of the listed EBPs and a further 15% only one or two. Thus, a considerable number of professionals did not use as many EBPs as they could. Limited resources, an underdeveloped health system; a lack of knowledge about ASD, and insufficient training may be reasons. A lack of knowledge, however, is unlikely, as all participants worked with children and adolescents with ASD. But, only 43% said they had the necessary resources and only 50% claimed to receive ongoing training. Other studies also reported these factors as reasons for inadequate access to treatments in LMIC including Bangladesh (Bangladesh Bureau of Statistics, 2018; Dababnah & Bulson, 2015).

Hypothesis 4 was that practitioners’ attitudes and demographic variables, as well as barriers, predict the use of EBPs. This hypothesis was partially supported. Only the requirement subscale and service setting were significant predictors of the number of different treatments used. One explanation why perceived barriers and attitudes were not predictive of the usage of EBPs in Bangladesh is that public health organizations have a strong formalization of rules, many regulations, and hierarchical authority structures. Thus, the types of treatments used are highly regulated. Our findings seem inconsistent with previous research (Aarons et al., 2009; Jensen-Doss et al., 2009; Reding et al., 2014) showing perceived barriers, limitations, and lack of appealing features to hinder the implementation and usage of EBPs. Note, however, that we did not investigate how often and how intensive participating professionals use EBPs, but how many different types they use. It could be that barriers and attitudes are not predictive of usage per se, but of the frequency and intensity with which the treatments are used.

### Limitations

There are some limitations that should be considered. First, the sampling procedure may have limited the representativeness of the sample. To be included, professionals had to be listed in one of the registries we assessed and had to be specialized on working with children with ASD and other NDDs. It turned out that the final sample only came from the area of Dhaka. Health care providers from rural areas were lacking from our sample. Therefore, the generalizability of the findings may be limited to the capital of Bangladesh.

A second limitation is the sample size. As outlined above, the number was sufficient to establish small to medium effects with respect to Hypotheses 1 and 2. Further analyses investigating the three different settings and differences within these settings, however, were not possible. Note that the overall number of eligible professionals was rather small, which reflects the situation in Bangladesh. There are only a few trained professionals caring for children with ASD. Of the eligible professionals, a large number participated (76%).

A third limitation is that the study considered only a limited set of factors potentially relevant for the implementation and usage of EBPs. The survey included demographical variables, attitudes, and perceived barriers, but not other aspects that are known to contribute to the use of EBPs in mental health settings, such as leadership, organizational climate, and system factors. As described above, the current survey already took 45 min to complete. Given the temporal constraints and the high workload of professionals in Bangladesh, we were not able to address more factors in the survey.

A fourth limitation was that we had to translate the EBPAS-36 (Rye et al., 2017) into Bangla for this study. Although the EBPAS-36 is a well-validated instrument in Western countries (e.g., Norway, USA, the Netherlands, and Germany), it has not been validated in the context of Bangladesh. Therefore, we cannot be sure that measurement invariance is given. The low internal consistency of some of the subscales may indicate respective problems. Note, however, that the internal consistencies of the subscales for which significant results were found, were good. Excluding the inconsistent subscales, would not have changed our results and the interpretation of the findings.

### Implications for Practice and Future Research

Despite the limitations, this study has important implications. The dissemination and implementation of EBPs to improve the quality of mental health services and outcomes for children with ASD is a relatively new field in Bangladesh and other LMICs. Increasing EBP delivery has the potential to enhance treatment outcomes for youth and families. A clearer understanding of the factors precipitating providers’ adoption of EBPs may help to bridge the evidence-practice gap when implementing EBPs. The current study’s findings may be helpful for the realization of the Bangladesh government’s plan to improve services and outcomes for children and adolescents with ASD and other NDDs. The results show the barriers that have to be addressed when implementing the strategic plan. Considering the rather low number of participants who receive ongoing training, the government and non-governmental organizations should develop certifying training programs, research programs, and treatment dissemination strategies for mental health professionals, special teachers, and associate service staff. These programs should increase the knowledge about EBPs and address concerns related to EBPs use.

Within organizations, conditions (e.g., support by supervisors, available resources, processes and systems) seem to support the usage of EBPs quite often, but not always. Therefore, these conditions should be explored in each organization, before the implementation of new EBPs starts. Implementation strategies should be tailored to the specific organization and its professionals to improve attitudes and thereby increase the usage of EBPs.

Parent-mediated interventions are being used in many low-resource settings, yet inequalities in access to these programs remain a challenge, due to financial, geographic, and cultural barriers (Gillespie-Lynch & Brezis, 2018). The same is true for Bangladesh. The findings indicate that 1 in 3 participating professionals did not use parent training. The use of existing parent-mediated ASD interventions, which are designed in HICs, is difficult and the materials usually require a great deal of modification and adaptation. The government should strengthen efforts towards increasing awareness of these interventions and support researchers, who are developing community-based ASD interventions in the context of Bangladesh.

The current research showed that attitudes towards EBP and perceived barriers varied between professionals and within different service settings. Therefore, it is important to do further research using mixed methods in the specific branches of the Bangladeshi health system as described above. Furthermore, future research should examine the extent to which patient outcomes vary depending on attitudes towards EBP, barriers, and implemented EBPs.

These recommendations may support the success of “The National Strategic Plan for Neuro-Developmental Disorders 2016–2021” by the Government of Bangladesh, which aims to spread the use of EBPs. We hope that the findings help to successfully implement EBPs, which reduce the disability and improve the quality of life of people with ASD.

## Data Availability

The data are available upon request from the first author.

## Footnotes

1 Evidence-based practice is the integration of best research evidence with clinical expertise in the context of patient characteristics, culture, and preferences (American Psychological Association, 2005, p. 5). Evidence-based practices (EBPs) are treatments or programs that have been proven efficacious through research (NPDC, 2017a; 2017b). Thus, EBP should use EBPs.

2 See section on current use of EBPs in the method section for details.

## References

[CR1] Aarons GA (2004). Mental health provider attitudes toward adoption of evidence-based practice: The Evidence-Based Practice Attitude Scale (EBPAS). Mental Health Services Research.

[CR2] Aarons GA (2005). Measuring provider attitudes toward evidence-based practice: Consideration of organizational context and individual differences. Child and Adolescent Psychiatric Clinics.

[CR3] Aarons GA, Cafri G, Lugo L, Sawitzky A (2012). Expanding the domains of attitudes towards evidence-based practice: The evidence-based practice attitude scale-50. Administration and Policy in Mental Health and Mental Health Services Research.

[CR4] Aarons GA, Glisson C, Hoagwood K, Kelleher K, Landsverk J, Cafri G (2010). "Psychometric properties and U.S. national norms of the Evidence-Based Practice Attitude Scale (EBPAS)": Correction to Aarons et al. (2010). Psychological Assessment.

[CR5] Aarons GA, Hurlburt M, Horwitz SM (2011). Advancing a conceptual model of evidence-based practice implementation in public service sectors. Administration and Policy in Mental Health and Mental Health Services Research.

[CR6] Aarons GA, Sommerfeld DH, Hecht DB, Silovsky JF, Chaffin MJ (2009). The impact of evidence-based practice implementation and fidelity monitoring on staff turnover: Evidence for a protective effect. Journal of Consulting and Clinical Psychology.

[CR7] Adugna MB, Nabbouh F, Shehata S, Ghahari S (2020). Barriers and facilitators to healthcare access for children with disabilities in low and middle income sub-Saharan African countries: A scoping review. BMC Health Services Research.

[CR8] Ahmed, S. M., Alam, B. B., Anwar, I., Begum, T., Huque, R., Khan, J. A. M., Nababan, H., & Osman, F. A. (2015). *Bangladesh health system review*. WHO Regional Office for the Western Pacific. https://iris.wpro.who.int/bitstream/handle/10665.1/11357/9789290617051_eng.pdf

[CR9] Ahmed SM, Hossain MA, RajaChowdhury AM, Bhuiya AU (2011). The health workforce crisis in Bangladesh: Shortage, inappropriate skill-mix and inequitable distribution. Human Resources for Health.

[CR10] Akhter S, Hussain AE, Shefa J, Kundu GK, Rahman F, Biswas A (2018). Prevalence of Autism Spectrum Disorder (ASD) among the children aged 18–36 months in a rural community of Bangladesh: A cross-sectional study. F1000Research.

[CR11] Alam F, Hossain R, Ahmed HU, Alam MT, Sarkar M, Halbreich U (2020). Stressors and mental health in Bangladesh: Current situation and future hopes. Bjpsych International.

[CR87] American Psychological Association. (2005). *Report of the 2005 Presidential Task Force on evidence-based practice*. https://www.apa.org/practice/resources/evidence/evidence-based-report.pdf

[CR12] American Psychiatric Association (2013). Diagnostic and statistical manual of mental disorders (DSM-5®).

[CR13] Bachmann CJ, Gerste B, Hoffmann F (2018). Diagnoses of autism spectrum disorders in Germany: Time trends in administrative prevalence and diagnostic stability. Autism.

[CR14] Bangladesh Bureau of Statistics. (2018). Statistics and Informatics Division (SID) & Ministry of Planning. http://bbs.portal.gov.bd/sites/default/files/files/bbs.portal.gov.bd/page/a1d32f13_8553_44f1_92e6_8ff80a4ff82e/Bangladesh%20%20Statistics-2018.pdf

[CR15] Beidas RS, Marcus S, Aarons GA, Hoagwood KE, Schoenwald S, Evans AC, Hurford MO, Hadley T, Barg FK, Walsh LM, Adams DR, Mandell DS (2015). Predictors of community therapists’ use of therapy techniques in a large public mental health system. JAMA Pediatrics.

[CR16] Blake JM, Rubenstein E, Tsai PC, Rahman H, Rieth SR, Ali H, Lee LC (2017). Lessons learned while developing, adapting and implementing a pilot parent-mediated behavioural intervention for children with autism spectrum disorder in rural Bangladesh. Autism.

[CR17] Boilson AM, Staines A, Ramirez A, Posada M, Sweeney MR (2016). Operationalization of the European Protocol for Autism Prevalence (EPAP) for autism spectrum disorder prevalence measurement in Ireland. Journal of Autism and Developmental Disorders.

[CR18] Buescher AV, Cidav Z, Knapp M, Mandell DS (2014). Costs of autism spectrum disorders in the United Kingdom and the United States. JAMA Pediatrics.

[CR19] Burgess AM, Okamura KH, Izmirian SC, Higa-McMillan CK, Shimabukuro S, Nakamura BJ (2017). Therapist attitudes towards evidence-based practice: A joint factor analysis. The Journal of Behavioral Health Services & Research.

[CR20] Ching H, Pringsheim T (2012). Aripiprazole for autism spectrum disorders (ASD). Cochrane Database of Systematic Reviews.

[CR21] Connors EH, Schiffman J, Stein K, LeDoux S, Landsverk J, Hoover S (2019). Factors associated with community-partnered school behavioral health clinicians’ adoption and implementation of evidence-based practices. Administration and Policy in Mental Health and Mental Health Services Research.

[CR22] Croen LA, Najjar DV, Ray GT, Lotspeich L, Bernal P (2006). A comparison of health care utilization and costs of children with and without autism spectrum disorders in a large group-model health plan. Pediatrics.

[CR23] Dababnah S, Bulson K (2015). “On the sidelines”: Access to autism-related services in the West Bank. Journal of Autism and Developmental Disorders.

[CR24] Damschroder LJ, Aron DC, Keith RE, Kirsh SR, Alexander JA, Lowery JC (2009). Fostering implementation of health services research findings into practice: A consolidated framework for advancing implementation science. Implementation Science.

[CR25] Dereu M, Warreyn P, Raymaekers R, Meirsschaut M, Pattyn G, Schietecatte I, Roeyers H (2010). Screening for autism spectrum disorders in Flemish day-care centres with the checklist for early signs of developmental disorders. Journal of Autism and Developmental Disorders.

[CR26] Directorate General of Health Services, Ministry of Health and Family Welfare, Bangladesh. (2016). *National Strategic Plan for Neurodevelopmental Disorders 2016–2021. *Retrieved April 1, 2020, from https://www.medbox.org/bd-policies-others/national-strategic-plan-for-neurodevelopmental-disorders-2016-2021-bangladesh/preview.

[CR27] Ehsan U, Sakib N, Haque MM, Soron T, Saxena D, Ahamed SI, Schwichtenberg A, Rabbani G, Akter S, Alam F, Begum A, Ahmed SI (2018). Confronting autism in urban Bangladesh: unpacking infrastructural and cultural challenges. EAI Endorsed Transactions on Pervasive Health and Technology.

[CR28] Eid AM, Aljaser SM, AlSaud AN, Asfahani SM, Alhaqbani OA, Mohtasib RS, Aldhalaan HM, Fryling M (2017). Training parents in Saudi Arabia to implement discrete trial teaching with their children with autism spectrum disorder. Behavior Analysis in Practice.

[CR29] Eisner D, Zoller M, Rosemann T, Huber CA, Badertscher N, Tandjung R (2011). Screening and prevention in Swiss primary care: A systematic review. International Journal of General Medicine.

[CR31] Fontaine P, Ross SE, Zink T, Schilling LM (2010). Systematic review of health information exchange in primary care practices. The Journal of the American Board of Family Medicine.

[CR32] Frambach RT, Schillewaert N (2002). Organizational innovation adoption: A multi-level framework of determinants and opportunities for future research. Journal of Business Research.

[CR33] Fung LK, Mahajan R, Nozzolillo A, Bernal P, Krasner A, Jo B, Coury D, Whitaker A, Veenstra-Vanderweele J, Hardan AY (2016). Pharmacologic treatment of severe irritability and problem behaviors in autism: A systematic review and meta-analysis. Pediatrics.

[CR34] Gillespie-Lynch, K., & Brezis, R. (2018). Parent-implemented interventions around the globe. In *Handbook of parent-implemented interventions for very young children with autism* (pp. 359–383). Springer. 10.1007/978-3-319-90994-3_22

[CR35] Glasman LR, Albarracin D (2006). Forming attitudes that predict future behavior: A meta-analysis of the attitude-behavior relation. Psychological Bulletin.

[CR36] Gliem, J. A., & Gliem, R. R. (2003). Calculating, interpreting, and reporting Cronbach’s alpha reliability coefficient for Likert-type scales. Midwest Research-to-Practice Conference in Adult, Continuing, and Community Education. http://hdl.handle.net/1805/344

[CR37] Glisson C, Schoenwald SK (2005). The ARC organizational and community intervention strategy for implementing evidence-based children's mental health treatments. Mental Health Services Research.

[CR38] Glisson, C., Schoenwald, S. K., Kelleher, K., Landsverk, J., Hoagwood, K. E., Mayberg, S., Green, P., & Research Network on Youth Mental Health (2008). Therapist turnover and new program sustainability in mental health clinics as a function of organizational culture, climate, and service structure. Administration and Policy in Mental Health and Mental Health Services Research.

[CR39] Global Autism Movement and Bangladesh. (2014)*. Center for Research and Information.* Retrieved September 3, 2014, from http://cri.org.bd/2014/09/03/global-autism-movement-and-bangladesh/.

[CR40] Greenhalgh T, Robert G, Macfarlane F, Bate P, Kyriakidou O (2004). Diffusion of innovations in service organizations: Systematic review and recommendations. The Milbank Quarterly.

[CR41] Grol R, Wensing M (2004). What drives change? Barriers to and incentives for achieving evidence-based practice. Medical Journal of Australia.

[CR42] Harrison AJ, Long KA, Manji KP, Blane KK (2016). Development of a brief intervention to improve knowledge of autism and behavioral strategies among parents in Tanzania. Intellectual and Developmental Disabilities.

[CR43] Hasan MT, Anwar T, Christopher E, Hossain S, Hossain MM, Koly KN, Saif-Ur-Rahman KM, Ahmed HU, Arman N, Hossain SW (2021). The current state of mental healthcare in Bangladesh: Part 1–an updated country profile. Bjpsych International.

[CR44] Hirsch LE, Pringsheim T (2016). Aripiprazole for autism spectrum disorders (ASD). Cochrane Database of Systematic Reviews.

[CR45] Holzer, P., Lewig, K., Bromfield, L., & Arney, F. (2007). Research use in the Australian child and family welfare sector.

[CR46] Hossain MD, Ahmed HU, Uddin MJ, Chowdhury WA, Iqbal MS, Kabir RI, Chowdhury IA, Aftab A, Datta PG, Rabbani G, Hossain SW, Sarker M (2017). Autism Spectrum disorders (ASD) in South Asia: A systematic review. BMC Psychiatry.

[CR47] Idring S, Lundberg M, Sturm H, Dalman C, Gumpert C, Rai D, Lee BK, Magnusson C (2015). Changes in prevalence of autism spectrum disorders in 2001–2011: Findings from the Stockholm youth cohort. Journal of Autism and Developmental Disorders.

[CR48] Iemmi, V., Knapp, M., & Ragan, I. (2017). The autism dividend: Reaping the rewards of better investment. National Autism Project.

[CR49] Jackson, S. L., Volkmar, F. R., & Volkmar, F. (2019). Diagnosis and definition of autism and other pervasive developmental disorders. *Autism and pervasive developmental disorders*, 1–24.

[CR50] Jensen-Doss A, Hawley KM, Lopez M, Osterberg LD (2009). Using evidence-based treatments: The experiences of youth providers working under a mandate. Professional Psychology: Research and Practice.

[CR51] Kauth MR, Sullivan G, Cully J, Blevins D (2011). Facilitating practice changes in mental health clinics: A guide for implementation development in health care systems. Psychological Services.

[CR52] Khaleghi A, Zarafshan H, Vand SR, Mohammadi MR (2020). Effects of non-invasive neurostimulation on autism spectrum disorder: A systematic review. Clinical Psychopharmacology and Neuroscience.

[CR53] Khan NZ, Sultana R, Ahmed F, Shilpi AB, Sultana N, Darmstadt GL (2018). Scaling up child development centres in Bangladesh. Child: Care, Health and Development.

[CR54] Kim DD, Silver MC, Kunst N, Cohen JT, Ollendorf DA, Neumann PJ (2020). Perspective and costing in cost-effectiveness analysis, 1974–2018. PharmacoEconomics.

[CR55] Lai MC, Baron-Cohen S (2015). Identifying the lost generation of adults with autism spectrum conditions. The Lancet Psychiatry.

[CR56] Lau R, Stevenson F, Ong BN, Dziedzic K, Treweek S, Eldridge S, Everitt H, Kennedy A, Qureshi N, Rogers A, Peacock R, Murray E (2015). Achieving change in primary care—Causes of the evidence to practice gap: Systematic reviews of reviews. Implementation Science.

[CR57] Lavrakas P (2008). Encyclopedia of survey research methods.

[CR58] Leonard E, de Kock I, Bam W (2020). Barriers and facilitators to implementing evidence-based health innovations in low-and middle-income countries: A systematic literature review. Evaluation and Program Planning.

[CR59] Locke J, Lawson GM, Beidas RS, Aarons GA, Xie M, Lyon AR, Stahmer A, Seidman M, Frederick L, Oh C, Spaulding C, Dorsey S, Mandell DS (2019). Individual and organizational factors that affect implementation of evidence-based practices for children with autism in public schools: A cross-sectional observational study. Implementation Science.

[CR60] Lyall K, Croen L, Daniels J, Fallin MD, Ladd-Acosta C, Lee BK, Park BY, Snyder NW, Schendel D, Volk H, Windham GC, Newschaffer C (2017). The changing epidemiology of autism spectrum disorders. Annual Review of Public Health.

[CR61] Maenner MJ, Shaw KA, Baio J (2020). Prevalence of autism spectrum disorder among children aged 8 years—autism and developmental disabilities monitoring network, 11 sites, United States, 2016. MMWR Surveillance Summaries.

[CR62] Mannan M (2017). Autism in Bangladesh: Current scenario and future prospects. European Journal of Paediatric Neurology.

[CR63] McPheeters ML, Warren Z, Sathe N, Bruzek JL, Krishnaswami S, Jerome RN, Veenstra-VanderWeele J (2011). A systematic review of medical treatments for children with autism spectrum disorders. Pediatrics.

[CR64] Michie S, Johnston M, Abraham C, Lawton R, Parker D, Walker A (2005). Making psychological theory useful for implementing evidence based practice: A consensus approach. BMJ Quality & Safety.

[CR65] Mullick MSI, Goodman R (2005). The prevalence of psychiatric disorders among 5–10 year olds in rural, urban and slum areas in Bangladesh. Social Psychiatry and Psychiatric Epidemiology.

[CR66] National Autism Center (2009). National standards report, phase 1.

[CR69] National Autism Center (2015). Findings and conclusions: National standards project, phase 2.

[CR68] National Professional Development Center on Autism Spectrum Disorder. (2017a). *Matrix of evidence-based practices by outcome and age*. The University of North Carolina, Frank Porter Graham Child Development Institute. https://autismpdc.fpg.unc.edu/implementation.

[CR67] National Professional Development Center on Autism Spectrum Disorder. (2017b). *Comparison of NPDC and NSP EBPs*. The University of North Carolina, Frank Porter Graham Child Development Institute. https://autismpdc.fpg.unc.edu/evidence-based-practices.

[CR70] National Research Council (2001). Educating children with autism.

[CR71] NCDC, R., & BMRC, D. Survey of Autism and Neurodevelopmental Disorders in Bangladesh, 2013. *Non Communicable Diseases Control (NCDC) Programme, DGHS, MOHFW, Revitalization of Community Health Care Initiatives in Bangladesh (RCHCIB), Ministry of health and family welfare (MOHFW)*.

[CR72] Odom SL, Collet-Klingenberg L, Rogers SJ, Hatton DD (2010). Evidence-based practices in interventions for children and youth with autism spectrum disorders. Preventing School Failure: Alternative Education for Children and Youth.

[CR73] Odom S, Hume K, Boyd B, Stabel A (2012). Moving beyond the intensive behavior treatment versus eclectic dichotomy: Evidence-based and individualized programs for learners with ASD. Behavior Modification.

[CR74] Okamura KH, Hee PJ, Jackson D, Nakamura BJ (2018). Furthering our understanding of therapist knowledge and attitudinal measurement in youth community mental health. Administration and Policy in Mental Health and Mental Health Services Research.

[CR75] Olusanya BO, Davis AC, Wertlieb D, Boo NY, Nair MKC, Halpern R, Kuper H, Breinbauer C, de Vries PJ, Gladstone M, Halfon N, Kancherla V, Mulaudzi MC, Kakooza-Mwesige A, Ogbo FA, Olusanya JO, Williams AN, Wright SM, Manguerra H, Whiteford HA, Olsen HE, Kassebaum NJ (2018). Developmental disabilities among children younger than 5 years in 195 countries and territories, 1990–2016: a systematic analysis for the Global Burden of Disease Study 2016. The Lancet Global Health.

[CR76] Pagoto SL, Spring B, Coups EJ, Mulvaney S, Coutu MF, Ozakinci G (2007). Barriers and facilitators of evidence-based practice perceived by behavioral science health professionals. Journal of Clinical Psychology.

[CR77] Parner ET, Thorsen P, Dixon G, De Klerk N, Leonard H, Nassar N, Bourke J, Bower C, Glasson EJ (2011). A comparison of autism prevalence trends in Denmark and Western Australia. Journal of Autism and Developmental Disorders.

[CR78] Parsons D, Cordier R, Vaz S, Lee HC (2017). Parent-mediated intervention training delivered remotely for children with autism spectrum disorder living outside of urban areas: Systematic review. Journal of Medical Internet Research.

[CR79] Patra S, Kar SK (2021). Autism spectrum disorder in India: A scoping review. International Review of Psychiatry.

[CR80] Pervin, M., Ahmed, H. U., & Hagmayer, Y. (2021). *Effectiveness of Interventions for Children and Adolescents with Autism Spectrum Disorder in High Income vs. Low-Middle Income Countries: An overview of systematic reviews and research papers from LMIC* [Manuscript submitted for publication]. Institute of Psychology, Georg August University of Goettingen. 10.3389/fpsyt.2022.834783 PMC9386527 35990045

[CR81] Powell BJ, Mandell DS, Hadley TR, Rubin RM, Evans AC, Hurford MO, Beidas RS (2017). Are general and strategic measures of organizational context and leadership associated with knowledge and attitudes toward evidence-based practices in public behavioral health settings? A Cross-Sectional Observational Study. Implementation Science.

[CR82] R Core Team. (2020). R: A language and environment for statistical computing. R Foundation for Statistical Computing. https://www.R-project.org/

[CR83] Rabbani MG, Alam MF, Ahmed HU, Sarkar M, Islam MS, Anwar N, Zaman MM, Chowdhury S, Chowdhury MWA, Das SK, Hamid MA, Islam MT, Mohit MA, Jahan NA, Rahman AHM, Chowdhury S, Chowdhury KP, Wahab MA, Rahman F, Mandal MC, Hossain MD, Bhowmik AD, Bashar MK, Khan NM, Uddin MJ, Khan MZR (2009). Prevalence of mental disorders, mental retardation, epilepsy and substance abuse in children. Bangladesh Journal of Psychiatry.

[CR84] Raghavan R, Bright CL, Shadoin AL (2008). Toward a policy ecology of implementation of evidence-based practices in public mental health settings. Implementation Science.

[CR85] Reding ME, Chorpita BF, Lau AS, Innes-Gomberg D (2014). Providers’ attitudes toward evidence-based practices: Is it just about providers, or do practices matter, too?. Administration and Policy in Mental Health and Mental Health Services Research.

[CR86] Reichow B, Servili C, Yasamy MT, Barbui C, Saxena S (2013). Non-specialist psychosocial interventions for children and adolescents with intellectual disability or lower-functioning autism spectrum disorders: a systematic review. PLoS Medicine.

[CR88] Rye M, Friborg O, Skre I (2019). Attitudes of mental health providers towards adoption of evidence-based interventions: Relationship to workplace, staff roles and social and psychological factors at work. BMC Health Services Research.

[CR89] Rye M, Torres EM, Friborg O, Skre I, Aarons GA (2017). The Evidence-based Practice Attitude Scale-36 (EBPAS-36): A brief and pragmatic measure of attitudes to evidence-based practice validated in US and Norwegian samples. Implementation Science.

[CR90] Saemundsen E, Magnússon P, Georgsdóttir I, Egilsson E, Rafnsson V (2013). Prevalence of autism spectrum disorders in an Icelandic birth cohort. British Medical Journal Open.

[CR91] Samms-Vaughan ME (2014). The status of early identification and early intervention in autism spectrum disorders in lower-and middle-income countries. International Journal of Speech-Language Pathology.

[CR92] Saraceno B, van Ommeren M, Batniji R, Cohen A, Gureje O, Mahoney J, Sridhar D, Underhill C (2007). Barriers to improvement of mental health services in low-income and middle-income countries. The Lancet.

[CR93] Saxena S, Thornicroft G, Knapp M, Whiteford H (2007). Resources for mental health: Scarcity, inequity, and inefficiency. The Lancet.

[CR94] Shaw KA, Maenner MJ, Baio J (2020). Early identification of autism spectrum disorder among children aged 4 years—Early Autism and Developmental Disabilities Monitoring Network, six sites, United States, 2016. MMWR Surveillance Summaries.

[CR95] Siegel M, Beaulieu AA (2012). Psychotropic medications in children with autism spectrum disorders: A systematic review and synthesis for evidence-based practice. Journal of Autism and Developmental Disorders.

[CR96] Skonieczna-Żydecka K, Gorzkowska I, Pierzak-Sominka J, Adler G (2017). The prevalence of autism spectrum disorders in West Pomeranian and Pomeranian regions of Poland. Journal of Applied Research in Intellectual Disabilities.

[CR97] Smith BD (2013). Substance use treatment counselors’ attitudes toward evidence-based practice: The importance of organizational context. Substance Use & Misuse.

[CR98] Steinbrenner, J. R., Hume, K., Odom, S. L., Morin, K. L., Nowell, S. W., Tomaszewski, B., Szendrey, S., Mclntyre, N. S., Yücesoy-Özkan, S., & Savage, M. N. (2020). Evidence-based practices for children, youth, and young adults with autism. *FPG Child Development Institute*. 10.1007/s10803-020-04844-2 PMC8510990 33449225

[CR99] Toth SL, Manly JT (2011). Bridging research and practice: Challenges and successes in implementing evidence-based preventive intervention strategies for child maltreatment. Child Abuse & Neglect.

[CR100] Tregnago MK, Cheak-Zamora NC (2012). Systematic review of disparities in health care for individuals with autism spectrum disorders in the United States. Research in Autism Spectrum Disorders.

[CR101] van Bakel MME, Delobel-Ayoub M, Cans C, Assouline B, Jouk PS, Raynaud JP, Arnaud C (2015). Low but increasing prevalence of autism spectrum disorders in a French area from register-based data. Journal of Autism and Developmental Disorders.

[CR102] Varcin KJ, Jeste SS (2017). The emergence of autism spectrum disorder (ASD): Insights gained from studies of brain and behaviour in high-risk infants. Current Opinion in Psychiatry.

[CR103] Vassos MV, Carroll MF (2016). Assessing attitudes toward evidence-based practices of workers supporting people with disabilities: A validation of the evidence-based practice attitudes scale. American Journal on Intellectual and Developmental Disabilities.

[CR104] Williams NJ, Beidas RS (2019). Annual research review: The state of implementation science in child psychology and psychiatry: A review and suggestions to advance the field. Journal of Child Psychology and Psychiatry.

[CR105] Wisdom JP, Chor KHB, Hoagwood KE, Horwitz SM (2014). Innovation adoption: A review of theories and constructs. Administration and Policy in Mental Health and Mental Health Services Research.

[CR106] Wong, C., Odom, S. L., Hume, K. A., Cox, A. W., Fettig, A., Kucharczyk, S., Brock, M. E., Plavnick, J. B., Fleury, V. P., & Schultz, T. R. (2014). *Evidence-based practices for children, youth, and young adults with autism spectrum disorder*. Frank Porter Graham Child Development Institute. https://autismpdc.fpg.unc.edu/how-do-i-find-out-more-about-ebps 10.1007/s10803-014-2351-z 25578338

[CR107] Wong C, Odom SL, Hume KA, Cox AW, Fettig A, Kucharczyk S, Brock ME, Plavnick JB, Fleury VP, Schultz TR (2015). Evidence-based practices for children, youth, and young adults with autism spectrum disorder: A comprehensive review. Journal of Autism and Developmental Disorders.

[CR110] World Bank. (2020). *Historical classification by income.* https://datahelpdesk.worldbank.org/knowledgebase/articles/906519

[CR108] World Health Organization. (2011). Mental health atlas. Geneva.

[CR109] World Health Organization. (2015). *Bangladesh health system review*. WHO Regional Office for the Western Pacific. https://apps.who.int/iris/handle/10665/208214

[CR111] World Health Organization (2019). International statistical classification of diseases and related health problems, 11th revision.

[CR112] Yu Y, Chaulagain A, Pedersen SA, Lydersen S, Leventhal BL, Szatmari P, Aleksic B, Ozaki N, Skokauskas N (2020). Pharmacotherapy of restricted/repetitive behavior in autism spectrum disorder: A systematic review and meta-analysis. BMC Psychiatry.

